# Idiopathic pneumoperitoneum, a possible complication of pure oxygen administration in a neonate with laryngomalacia: a case report

**DOI:** 10.1097/RC9.0000000000000800

**Published:** 2026-08-12

**Authors:** Florent Tshibwid A Zeng, Yannick Kizonde Kalungwe, Mildred Chola Chembo, Mika Mulewa Kalumba, Yves Mukalay Banza, Catherine Saleh Ugumba

**Affiliations:** a Department of Surgery, Faculty of Medicine, Université de Lubumbashi, Lubumbashi, Democratic Republic of the Congo; b Department of Surgery, University Clinics of Lubumbashi, Lubumbashi, Democratic Republic of the Congo; c Department of Specialties, Faculty of Medicine, Université de Lubumbashi, Lubumbashi, Democratic Republic of the Congo; d Department of ENT, University Clinics of Lubumbashi, Lubumbashi, Democratic Republic of the Congo; e Department of Pediatrics, University Clinics of Lubumbashi, Lubumbashi, Democratic Republic of the Congo; f Department of Medical Imaging, University Clinics of Lubumbashi, Lubumbashi, Democratic Republic of the Congo; g Department of Anesthesia and Resuscitation, Faculty of Medicine, Université de Lubumbashi, Lubumbashi, Democratic Republic of the Congo; h Department of Anesthesia and Resuscitation, University Clinics of Lubumbashi, Lubumbashi, Democratic Republic of the Congo

**Keywords:** case report, laryngomalacia, neonate, oxygen, pneumoperitoneum

## Abstract

**Introduction and clinical importance:**

Idiopathic pneumoperitoneum can occur in a full-term neonate due to the administration of pure oxygen. Recognition of this rare mechanism is necessary to avoid unnecessary surgical exploration.

**Case presentation:**

We report the case of a six-day-old male neonate with a history of respiratory distress and non-invasive pure oxygen support (FiO_2_ = 1.00) via a nasal cannula. He progressively developed abdominal distension, but had no fever, abdominal tenderness, or redness. A massive pneumoperitoneum was noted on a plain X-ray. While preparing for surgical exploration, the distension regressed, and idiopathic pneumoperitoneum was diagnosed and managed conservatively. The respiratory distress was caused by laryngomalacia, which was also managed conservatively, with no complications noted 6 months after admission.

**Clinical discussion:**

Idiopathic pneumoperitoneum should be suspected in neonates with a history of respiratory distress but without classic clinical signs of peritonitis. Notably, noninvasive administration of pure oxygen alone can cause idiopathic pneumoperitoneum.

**Conclusion:**

In a full-term neonate, non-invasive administration of pure oxygen for laryngomalacia can cause idiopathic pneumoperitoneum, which may have a favorable outcome with conservative management.

## Introduction

Pneumoperitoneum, the presence of free air in the peritoneal cavity, occurs in approximately 0.6% of neonatal intensive care unit admissions^[^1^]^. In 90% of cases, it arises from gastrointestinal tract (GIT) perforation, a surgical emergency frequently caused by necrotizing enterocolitis (NEC), gastric perforation, Hirschsprung’s disease, or intestinal atresia^[^2,3^]^. Conversely, cases without GIT perforation are classified as spontaneous or idiopathic pneumoperitoneum (IP), representing 6–10% of cases^[^2^]^. IP primarily affects premature neonates undergoing mechanical ventilation (MV) for respiratory distress (RD) syndrome, in which high pressures can cause alveolar rupture^[^2–4^]^. Importantly, IP secondary to the noninvasive administration of pure oxygen has not been described in the literature. Unlike secondary pneumoperitoneum (SP), IP is conventionally managed conservatively^[^4^]^.

HIGHLIGHTS

- What is known about the subject: Idiopathic pneumoperitoneum is mainly encountered in preterm or low-birth-weight neonates who require oxygen support through mechanical ventilation due to neonatal respiratory distress syndrome.
- What this study adds: We report a case of a full-term, normal-weight neonate with no history of mechanical ventilation who received non-invasive administration of pure oxygen for a rare cause of respiratory distress: laryngomalacia.

This case has been reported in line with the 2025 SCARE checklist^[^5^]^. It details a full-term neonate presenting with IP via a rare, unrecorded mechanism (see Supplemental Digital Content 1, available at: https://links.lww.com/IJSCR/A107 for the timeline). Given that neonatal surgical exploration carries a significant risk of morbidity and mortality, understanding this peculiar disease presentation is crucial. This case serves to guide clinicians and surgeons in early identification, ultimately preventing unwarranted surgical interventions.

## Case presentation

A 6-day-old, full-term male neonate (birth weight: 3500 g) was transferred to pediatric surgery for progressive abdominal distension and fever (38.9°C). Born to a 28-year-old mother following an unremarkable pregnancy, the patient had no history of delayed meconium passage or resuscitation. He had experienced severe RD since day 1 of life, which worsened during feeding. This required admission to the pediatric intensive care unit and support with 1 L/min of pure oxygen via a nasal cannula. Initial chest radiographs and echocardiograms were unremarkable. Enteral feeding via a nasogastric tube was initiated on day 3. By day 6 (5 days since oxygen support had been started), the patient developed fever and marked abdominal distension despite passing loose stools. NEC was suspected, empirical antibiotics (cefotaxime and gentamicin) were initiated, and the patient was transferred.

**Figure 1.**
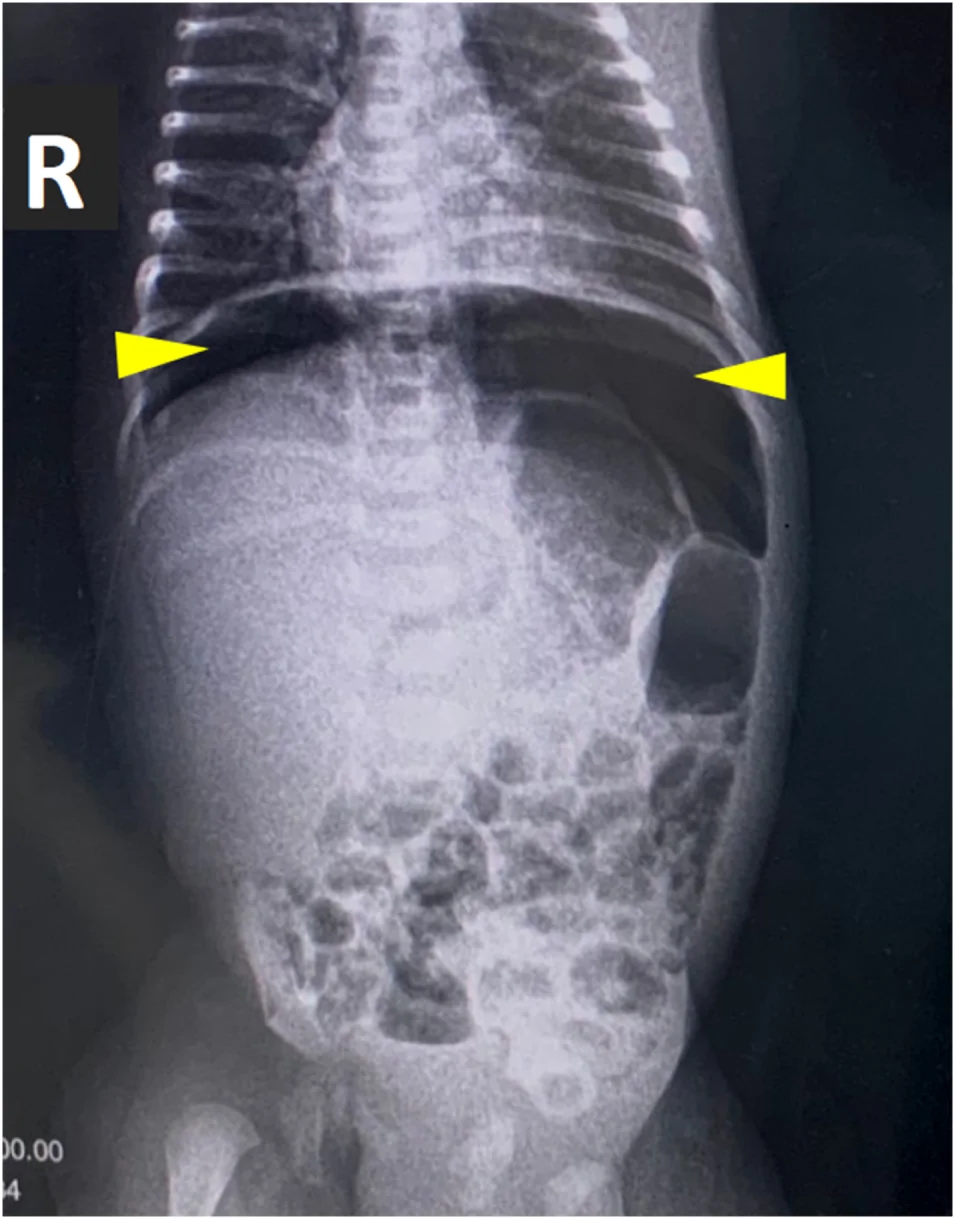
Plain abdominal x-ray. Note significant pneumoperitoneum under both sides of the diaphragmatic domes (yellow arrows) and no pulmonary disease.

**Figure 2.**
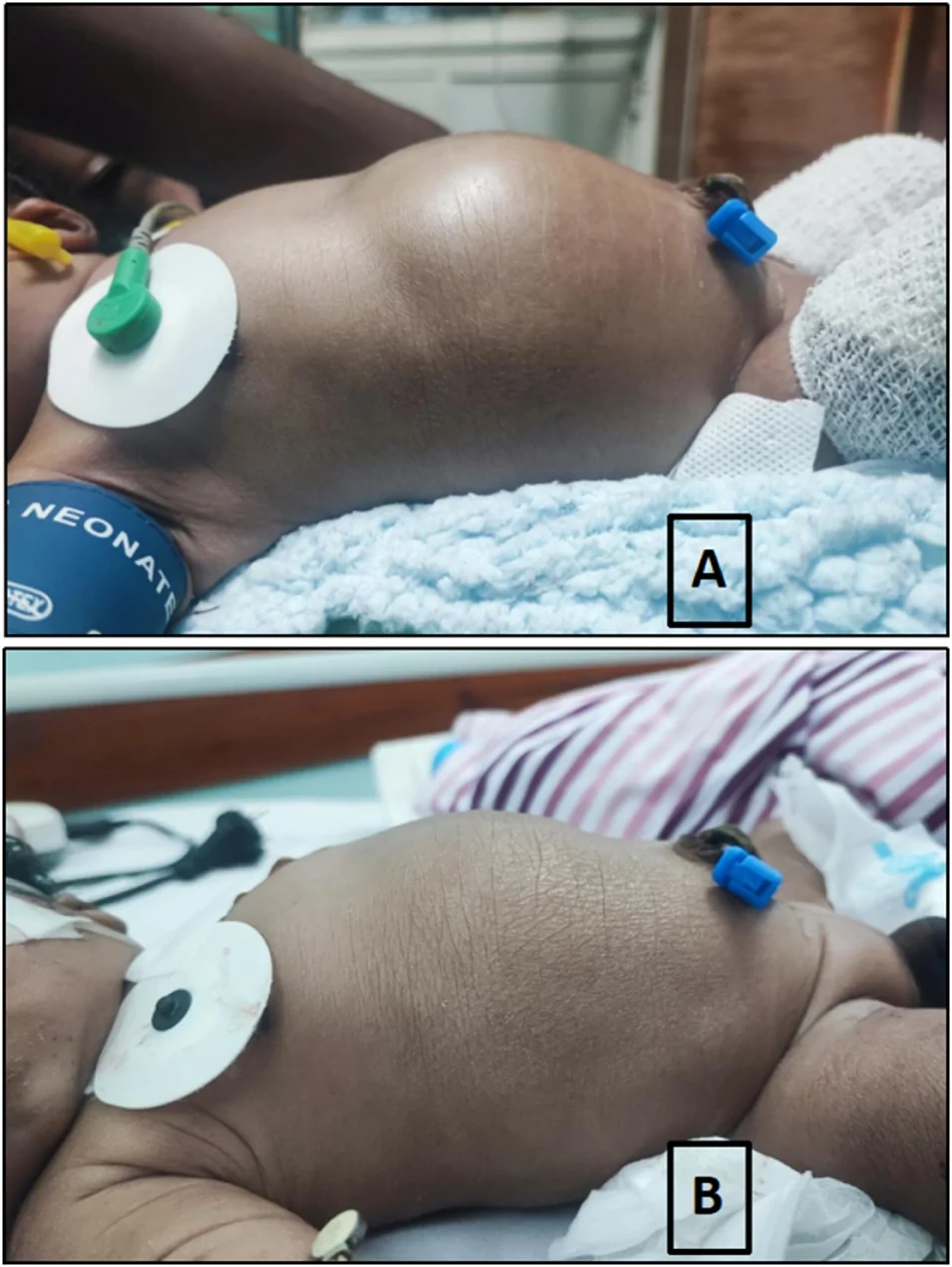
Clinical evolution within 5 hours. Note regression of abdominal distension from 45 cm (A) to 32 cm (B).

**Figure 3.**
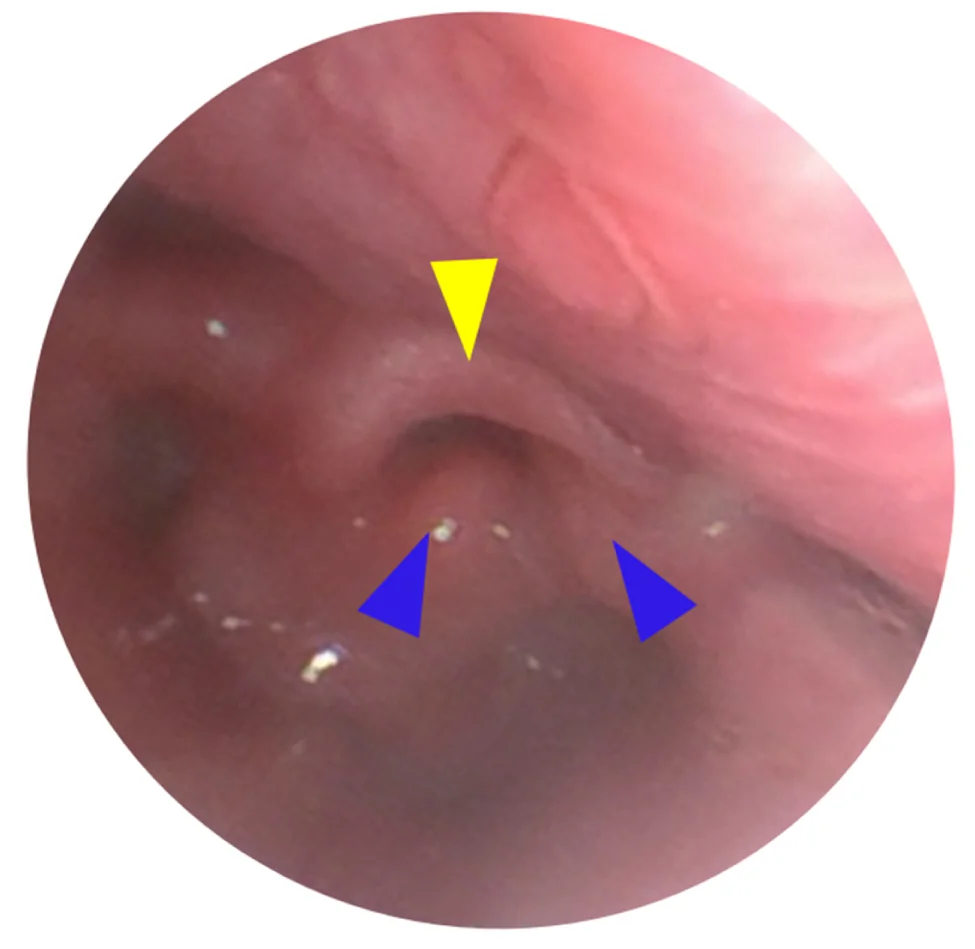
Nasolaryngoscopic findings. Note the inward collapse of the floppy supra-arytenoid tissues (blue arrows), with a normal shape of the epiglottis (yellow arrow).

Upon admission to the pediatric surgery service, the neonate was febrile at 38.5°C (which was initially interpreted as a sign of infection), tachycardic, tachypneic, and oxygen-dependent. Physical examination revealed a soft, non-erythematous abdomen, distended to 45 cm, with collateral venous circulation. Laboratory workup showed hyperleukocytosis (27 100/μL), elevated C-reactive protein (16 mg/dL), and normal platelets (325 000/μL). An abdominal radiograph demonstrated massive pneumoperitoneum (Fig. 1), prompting preparation for an emergency laparotomy for suspected gastrointestinal tract perforation. Within 5 hours of admission and during surgical preparation, the patient’s abdominal distension completely and spontaneously regressed (Fig. 2). The fever resolved, bowel sounds returned, and no bloody stools or emesis were observed. Consequently, a diagnosis of surgical perforation or NEC was excluded, and IP secondary to respiratory pathology was suspected. To investigate the underlying etiology of RD, an upper gastrointestinal series with diluted Gastrografin was performed, ruling out tracheoesophageal fistula, laryngotracheal cleft, or pneumomediastinum (see Supplemental Digital Content Material 2, available at: https://links.lww.com/IJSCR/A107). Subsequent fiberoptic nasolaryngoscopy revealed inspiratory collapse of floppy suprarytenoid tissues, confirming Olney type I laryngomalacia^[^6^]^ (Fig. 3).

The patient was transferred to the ENT department for conservative management, which included oral ondansetron, dexamethasone, and calcium supplementation. Oxygen support was successfully weaned within 24 hours, coinciding with complete resolution of RD. The patient was discharged 48 hours after weaning. At the 6-month follow-up visit, the infant remained completely asymptomatic, with normal growth.

The family's belief was that RD resulted from improper neonatal nasal aspiration after birth. Their concerns were mainly about the patient's postoperative survival. They expected rapid management and discharge of the patient to avoid the high cost of care.

## Discussion

We report a case of IP following non-invasive pure-oxygen administration for RD caused by type 1 laryngomalacia. Conservative management of this full-term patient resulted in a favorable outcome.

### Epidemiology

IP is exceedingly rare, representing < 10% of all neonatal pneumoperitoneum cases^[^2^]^. It shows no sex predilection but primarily affects preterm neonates (62–76%) and low-birth-weight infants (75%)^[^3,4^]^. Most affected patients have a history of mechanical or bag ventilation^[^3^]^, with RD typically due to neonatal RD syndrome. Less commonly reported causes include congenital heart disease, pneumonia, and surgery for syngnathia^[^2,7,8^]^. In contrast, this patient was a full-term neonate with a normal birth weight. The underlying cause of RD was laryngomalacia, which typically presents with stridor^[^6^]^; however, the absence of this classic sign in this patient reduced the initial index of suspicion. Notably, laryngomalacia has not been previously reported in patients with IP.

### Pathophysiology and proposed mechanism

In MV, pressure induces alveolar rupture in fragile preterm neonates. Free air travels along vascular sheaths to the hilum, creating pneumomediastinum or pneumothorax, before migrating through diaphragmatic orifices into the abdomen^[^4^]^.

Because MV was not used, pure oxygen administration was suspected. Hyperoxia increases mitochondrial reactive oxygen species (ROS) production, causing bioenergetic failure^[^9,10^]^. Excess ROS dysregulates the Nrf2-Keap1, NF-κB, and p53 pathways, inducing premature cell death via mitochondrial membrane permeabilization^[^10^]^. This permits cytochrome c leakage into the cytosol, initiating apoptosis^[^10^]^. The resulting alveolar membrane rupture releases free air, which replicates the anatomical migration pathways of MV-induced injury, producing pneumoperitoneum.

### Diagnostic differentiation

In IP, history and clinical examination raise suspicion. Most patients with IP are preterm, with previous RD and prior use of MV^[^3,7^]^. On physical examination, the general condition can vary from stable to very poor, especially in cases of comorbidity^[^5^]^. On abdominal examination, there is no classic redness of the SP and no tenderness^[^3,4^]^. Chest examination is essential and can reveal features of pneumothorax. On an erect plain X-ray, the association of pneumothorax or pneumomediastinum with pneumoperitoneum is highly suggestive^[^3,4^]^. In our patient, the atypical presentation decreased the index of suspicion for IP, which led to a misleading initial diagnosis and prompted an unnecessary emergency surgical intervention. Following regression of abdominal distension (possible exteriorization through the separating umbilical cord), the diagnosis of IP was confirmed.

### Management

The management of IP is mainly conservative. The primary management of the condition leading to RD results in resolution of IP^[^4^]^. Authors have reported abdominal drain placement to deflate abdominal distension; however, outcomes were unfavorable due to sepsis or congenital heart defects^[^4^]^. In cases where diagnostic doubt persists or the diagnosis is missed, laparotomy has been reported and was not associated with increased mortality^[^6^]^. In their review, *Kim et al* found 100% mortality associated with concurrent complications^[^3^]^. In our patient, we opted for conservative management of IP. The management of laryngomalacia depends on the severity of obstruction, as Olney’s types I and II spontaneously improve with age and conservative treatment^[^6^]^. Given moderate symptoms, conservative management was maintained in our patient, with clinical improvement.

### Strengths and limitations

The strengths of this case are the avoidance of surgical exploration and the identification of the noninvasive administration of pure oxygen as a possible cause of IP. The main limitation of this work is the presentation of a single case, which reduces its generalizability.

## Conclusion

When dealing with pneumoperitoneum without digestive symptoms in a neonate receiving pure oxygen, one should think of IP. However, rarely, it can occur in a full-term neonate associated with the administration of pure oxygen in the context of laryngomalacia. With conservative management, a good outcome is possible. Therefore, in stable patients with suspected IP, conservative management is warranted.

## Data Availability

Not applicable.

## Patient perspective

The patient’s parents were satisfied with the successful non-operative management of their child's condition with regression of RD.
